# Reduced cholesterol is associated with the depressive-like behavior in rats through modulation of the brain 5-HT1A receptor

**DOI:** 10.1186/s12944-015-0020-7

**Published:** 2015-03-24

**Authors:** Shuqin Sun, Shuo Yang, Yongjun Mao, Xiujuan Jia, Zheng Zhang

**Affiliations:** Department of Geriatric Medicine, The Affiliated Hospital of Qingdao University, 16, Jiangsu Road, Qingdao, Shandong Province 266000 China; Department of the Intensive Care Unit, The Affiliated Hospital of Qingdao University, 16, Jiangsu Road, Qingdao, Shandong Province 266000 China

**Keywords:** Cholesterol, Depression, Serotonin, Chronic stress, Medial prefrontal cortex

## Abstract

**Background:**

Low serum cholesterol levels are related to an increased risk of depression and its serious consequences. However, the effect of central cholesterol on depressive disorder and its potential regulatory mechanism is poorly understood. Therefore, brain cholesterol in patients with depression may not only decrease the risk for developing this disease but also increase the beneficial effects of treatment for depression.

**Methods:**

In current study, rats were exposed to chronic mild stress (CMS) for consecutive 28 days, and the depressive-like behavior was tested by sucrose preference test, immobility in the forced swim test, locomotor activity in the open field test, decreased bodyweight and food intake. Additionally, the total cholesterol levels in the medial prefrontal cortex (mPFC) and the hippocampus of rats were measured by gas chromatograph mass spectrometer. Finally, 5-HT1A receptor antagonist WAY100635 was used to determine the potential role of serotonin system in the interaction between central cholesterol and depression.

**Results:**

CMS significantly reduced total cholesterol levels in the mPFC but not in the hippocampus and resulted in depressive-like behavior. Chronic supplementation of cholesterol by food reversed the depressive-like behavior induced by CMS. Furthermore, pre-injection of 5-HT1A receptor antagonist WAY100635 into the mPFC blocked the treatment effects of cholesterol on the reversal of behavioral response.

**Conclusion:**

This finding suggested that cholesterol in the mPFC may have an impact on the sensitivity of the 5-HT1A receptor in the development and treatment of depression. The treatment benefits of cholesterol could be through modulation of the brain 5-HT1A receptor.

## Introduction

Individuals with serious mental illness are at increased risk for coronary artery disease. Acute coronary syndrome significantly decreases total cholesterol levels [1]. Aberrant lipid metabolism has also been observed in coronary artery disease patients with or without depression [2,3], as well as those with depression who are otherwise medically healthy [4,5], suggesting that lipid signaling plays a key role in the comorbidity. Patients with major depressive disorder (MDD) may have significant differences in cholesterol levels compared to healthy controls [6]. Accumulating evidence suggests that low serum total cholesterol levels related to an increased risk of depression and its serious consequences [7]. Most recently, low serum cholesterol levels have also been associated with suicidal behavior [8-11]. However, the effect of central cholesterol on depressive disorder is poorly understood.

It has been proposed that brain synaptosomal membrane cholesterol might determine the number of serotonin receptors [8]. Low membrane cholesterol decreases the number of serotonin receptors. Therefore, disturbed cholesterol levels may be associated with serotonergic dysfunction. There are ample evidences for a critical involvement of serotonergic receptors, particularly 5-HT1A, in the pathophysiology and treatment of depression [12,13]. In preclinical studies, 5-HT1A agonists increase prefrontal dopamine release [14,15]. As a consequence, the 5-HT1A receptor serves as an important target in the development of therapeutic agents for neuropsychiatric disorders such as anxiety and depression [16]. Previous study has demonstrated the requirement of membrane cholesterol in the organization, dynamics, and function of the 5-HT1A receptor [17,18]. Additionally, it has been shown that there is a significant reduction in the level of specific ligand binding and G-protein coupling to 5-HT1A receptors upon chronic cholesterol depletion, although the membrane receptor level is not reduced at all [18]. Therefore, we hypothesized that 5-HT1A receptors could be involved in the induction of depression by chronic mild stress through cholesterol reduction. Recent neuroimaging and medial post-mortem studies showed altered activity of discrete regions within the hippocampus and prefrontal cortex (mPFC) and their atrophy are associated with depression [19-21]. In addition, 5-HT1A heteroreceptors are abundantly expressed post-synaptically in the PFC, amygdala, and hippocampus to mediate serotonin actions on fear, anxiety, stress, and cognition, indicating a primary role of the 5-HT-PFC circuitry in effective treatment of depression [22-24].

Based on these findings, the present study examines whether the changes of brain cholesterol levels are involved in the depressive symptoms in chronic mild stress procedure and the modulation of 5-HT system in the prefrontal cortex. Furthermore, another aim of this study is to investigate the possibility that the combined administration of 5-HT1A receptor antagonist and cholesterol supplementation reverses the induction of depressive symptoms by chronic mild stress.

## Methods

### Animals and diets

Male Sprague-Dawley rats that weighed 200-220 g upon arrival were individually housed under a constant temperature (23 ± 2°C) and a 12 h/12 h light/dark cycle with free access to food and water before the initiation of the experiment. The experimental diet for supplementary cholesterol was provided by the experimental animal centre of our hospital with 1 g/kg of cholesterol and sufficient amounts of protein, minerals and vitamins for healthy maintenance. All of the animal procedures were performed in accordance with the National Institutes of Health Guide for the Care and Use of Laboratory Animals, and the procedures were approved by the Local Animal Use Committee. All of the behavioral tests and drug administrations were performed in the dark phase.

### Drugs

The selective 5-HT1A receptors antagonist WAY100635 maleate (Sigma-Aldrich, St. Louis, MO, USA) was dissolved in saline (0.9% NaCl). The solutions were prepared immediately before the stress and protected from the light during the experimental sessions. The dose of WAY100635 used for microinjection was selected as 40 nmol/μl based on previous reports [25].

### Intracerebral cannula implantation and intracranial injections

The rats were anesthetized with sodium pentobarbital (60 mg/kg, i.p.), and guide cannulae (23-gauge, Plastics One, Roanoke, VA, USA) were placed at a 23° angle toward the midline and implanted bilaterally 1 mm above the medial prefrontal cortex with the following stereotaxic coordinates: anterior/posterior (A/P), -3.2 mm; medial/lateral (M/L), ± 2.5 mm; dorsal/ventral (D/V), -3.3 mm [26,27]. Vehicle, or WAY100635 was intracranially microinjected using 10 μl Hamilton syringes (Hamilton, Reno, NV, USA) that were connected via polyethylene-50 tubing to 30-gauge injectors (Plastics One). A total volume of 0.5 μl was infused into each side over 1 min, and the injection syringe was left in place for an additional 1 min to allow for diffusion. At the end of the experiments, the rats were anesthetized with sodium pentobarbital (100 mg/kg, i.p.) and transcardially perfused. Cannula placements were assessed using Nissl staining with a thickness of 30 μm under light microscopy. Subjects with misplaced cannulae and pathological damage were excluded from the statistical analysis.

### Behavioral tests

#### Chronic mild stress protocol

The chronic mild stress protocol was adapted from previous study [28]. Briefly, rats were subjected to different mild stressors for 28 days: Day 1 (cold immobilization for 1 h at 4°C, tilted cages 45° for 24 h), Day 2 (immobilization for 1 h, crowding for 24 h), Day 3 (forced cold swim for 5 min, soiled bedding for 24 h), Day 4 (immobilization for 1 h, vibration for 1 h), Day 5 (tilted cages 45° for 24 h, cold immobilization for 1 h at 4°C), Day 6 (forced cold swim for 5 min at 4°C, crowding for 24 h), and Day 7 (vibration for 1 h, soiled bedding for 24 h). This schedule was repeated three more times (Table 1). Control rats were handled daily without any stress in the housing room.

**Table 1:** **The protocol and stress procedures for chronic mild stress**

**Date**	**Stressor**
Monday	Cold immobilization for 1 h at 4°C, tilted cages 45° for 24 h
Tuesday	Immobilization for 1 h, crowding for 24 h
Wednesday	Forced cold swim for 5 min, soiled bedding for 24 h
Thursday	Immobilization for 1 h, vibration for 1 h
Friday	Tilted cages 45° for 24 h, cold immobilization for 1 h at 4°C
Saturday	Forced cold swim for 5 min at 4°C, crowding for 24 h
Sunday	Vibration for 1 h, soiled bedding for 24 h

#### Sucrose preference test

The measurement of sucrose preference was performed as previously described [29]. The rats were trained to adapt to a 1% sucrose solution (w/v) for 48 h at the beginning of the experiment, during which two bottles of 1% sucrose solution were placed in each cage. After adaptation, rats were deprived of water and food for 24 h, followed by the sucrose preference test, in which the rats were housed in individual cages for 4 h and had free access to two bottles that contained 1% sucrose or tap water. The bottles were counterbalanced across the left and right sides of the cages throughout the experiment. The position of the two bottles was varied every 2 h during the test. At the end of 4 h, sucrose and water consumption (in milliliters) was measured, and sucrose preference (%) was calculated as the ratio of sucrose consumption to sucrose plus water consumption.

#### Forced swim test

The rats were placed in a 25 cm diameter × 65 cm height plastic cylinder that was filled to a depth of 30 cm with 23-25°C water for 15 min. The rats were tested 24 h later. Immobility was defined as the minimum movement required to passively keep the animal’s head above the water without other motions. The results are expressed as the time (in seconds) that the animals spent immobile during the 5 min test [30].

#### Open field test

The open field test was used to measure locomotor activity as previously described [31]. Briefly, the apparatus consisted of a 75 cm × 75 cm × 40 cm square arena divided into 25 equal squares (15 cm × 15 cm) on the floor of the arena. A single rat was placed in the center of the apparatus, and the number of crossings (i.e., entries into adjacent squares) was counted for 5 min.

### Measurement of cholesterol levels

Rats were killed by decapitation after behavioral tests. Their brains were quickly removed, dissected on ice and collected for subsequent cholesterol analysis. Prior to analysis, the brain samples were spun in a speed vacuum dryer (12 mbar; Savant AES 1000) for 24 h in order to express the individual sterol concentrations relative to the dry weight. The sterols were extracted from the dried tissue by placing in a 1.5 ml mixture of chloroform/methanol (at a 2:1 ratio) for 24 h at 4°C. Sterol levels were determined by a gas chromatograph mass spectrometer as described previously [32,33]. The blood samples were kept at room temperature for 1 h and then centrifuged at 3000 rpm for 10 min. The serum supernatant fraction was stored in another tube for the subsequent cholesterol assays.

### Tissue sample preparation

The rats were decapitated, and the brains were extracted based on our previous study [34]. Subsequently, bilateral tissue punches of the prefrontal cortex and hippocampus (16 gauge) were obtained from approximately 1 mm thick coronal sections cut in a Reichert-Jung 2800 Frigocut E cryostat at -20°C. The rostral faces of the coronal sections were approximately 3.2 mm for mPFC and -3.8 mm for hippocampus from bregma.

### Intracerebral cannula implantation and intracranial injections

The rats were anesthetized with sodium pentobarbital (60 mg/kg, i.p.), and guide cannulae (23-gauge, Plastics One, Roanoke, VA, USA) were placed at a 23° angle toward the midline and implanted bilaterally 1 mm above the medial prefrontal cortex with the following stereotaxic coordinates: anterior/posterior (A/P), -3.2 mm; medial/lateral (M/L), ± 2.5 mm; dorsal/ventral (D/V), -3.3 mm [26,27]. Vehicle or WAY100635 (40 nmol/μl) was intracranially microinjected using 10 μl Hamilton syringes (Hamilton, Reno, NV, USA) that were connected via polyethylene-50 tubing to 30-gauge injectors (Plastics One). A total volume of 0.5 μl was infused into each side over 1 min, and the injection syringe was left in place for an additional 1 min to allow for diffusion. At the end of the experiments, the rats were anesthetized with sodium pentobarbital (100 mg/kg, i.p.) and transcardially perfused. Cannula placements were assessed using Nissl staining with a thickness of 30 μm under light microscopy. Subjects with misplaced cannulae were excluded from the statistical analysis.

### Experimental design

#### Experiment 1: effects of chronic mild stress on the cholesterol levels in the brain

In this section, we used two groups of rats (*n* = 8 per group) to observe the possible impact of chronic mild stress on the central levels of cholesterol. One group of rats was subjected to chronic mild stress procedure for consecutive 28 days, while the other group rats were handled normally without any stress during the period. One day after the last stressor on day 29, rats were weighed, and the sucrose preference test, open field test, forced swim test and food intake in 1 h were measured consequently. After the behavioral tests, rats were killed by decapitation, and brain tissues of medial prefrontal cortex (mPFC) and hippocampus were collected for further cholesterol assay.

#### Experiment 2: effects of cholesterol supplementation on the depressive-like behavior

The purpose of this experiment was to investigate whether chronic cholesterol supplementation by daily diets reverses depressive-like behavior induced by chronic mild stress. Four groups of rats (*n* = 8 per group) were used in a 2 (control and CMS) × 2 (vehicle and cholesterol) factorial design to test the effect of cholesterol supplementation on the depressive-like behavior. Rats were subjected to CMS or control without any stress for 28 days and received vehicle or cholesterol on the beginning of the stress period. On day 29, the bodyweight was measured, the sucrose preference test, open field test, forced swim test and food intake in 1 h were performed to determine the possible treatment effects of chronic supplementation of cholesterol.

#### Experiment 3: role of chronic cholesterol treatment on the cholesterol content in serum and brain

We further aimed to explore whether cholesterol supplementation alters cholesterol content differentially in medial prefrontal cortex and hippocampus. Four groups of rats (*n* = 8 per group) were used in a 2 (control and CMS) × 2 (vehicle and cholesterol) factorial design to test the effect of supplementation on the cholesterol level. Rats were subjected to CMS or control for 28 days and received vehicle or cholesterol on the initiation of the stress or control period. On day 29, rats were killed by decapitation without behavioral tests, subsequently blood and brain tissues of mPFC and hippocampus were collected for further cholesterol assay.

#### Experiment 4: role of 5-HT1A receptor on the behavioral responses of cholesterol

To determine whether 5-HT system is involved in the treatment effects of cholesterol, eight groups of rats (*n* = 8) were used for either control or CMS, either vehicle or cholesterol, and either vehicle or WAY100635, respectively. Rats were micro-injected with 5-HT1A receptor antagonist WAY100635 (40 nmol/μl per side) or vehicle 1 h before daily cholesterol provision during the 28-day chronic mild stress process. On day 29, the bodyweight was measured, the sucrose preference test, open field test, forced swim test and food intake in 1 h were performed to determine the regulatory role of 5-HT1A receptor on the behavioral responses of cholesterol.

### Data analysis

The data are expressed as mean ± SEM and were analyzed using one- or two-way analysis of variance (ANOVA) followed by Tukey’s *post hoc* test (for details, see Results section). In the two-way ANOVA tests, two factors are involved: 1. CMS and non-CMS groups, 2. diet with and without cholesterol treatment. Values of P < 0.05 were considered statistically significant.

## Results

### Chronic mild stress induced depressive-like behavior in rats

**Figure 1:**
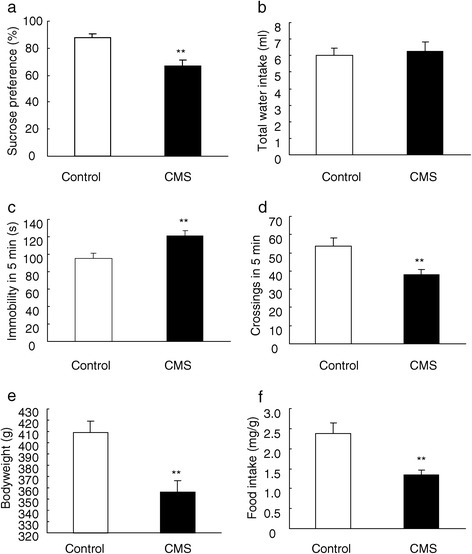
**Chronic mild stress induced depressive-like behavior in rats.** The behavioral tests were conducted after 28-day chronic mild stress. **(a)** sucrose preference, **(b)** total water intake, **(c)** immobility in the forced swim test, **(d)** locomotor activity in the open field test, **(e)** bodyweight, **(f)** food intake. Data are expressed as mean ± SEM (*n* = 8 per group). Differences between control and CMS were assessed using Tukey’s *post hoc* test. **P < 0.01 compared with control rats. CMS, chronic mild stress.

Rats subjected to chronic mild stress for 28 days, one of the most valid models of depression, showed anhedonia state as demonstrated by the decrease in sucrose preference (P < 0.01, Figure 1a) with on effects on the total water intake (Figure 1b) compared with rats in control group. In the forced swim test, the immobility was significantly increased by chronic mild stress procedure (P < 0.01, Figure 1c), suggesting a behavioral despair induced by chronic stress. Additionally, chronic mild stress reduced the locomotor activity of rats in the open field test (P < 0.01, Figure 1d). Furthermore, we also found that chronic mild stress decreased both the bodyweight (P < 0.01, Figure 1e) and the food intake (P < 0.01, Figure 1f) compared with control rats. These behavioral results suggest that chronic mild stress induced depressive-like behavior in rats.

### Chronic mild stress decreased cholesterol levels

**Figure 2:**
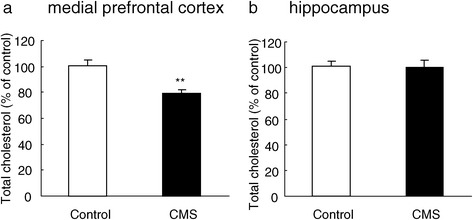
**Chronic mild stress decreased cholesterol levels.** The levels of cholesterol were significantly decreased by CMS in the medial prefrontal cortex **(a)** but not in the hippocampus **(b).** Data are expressed as mean ± SEM (*n* = 8 per group). Differences between control and CMS were assessed using Tukey’s *post hoc* test. **P < 0.01 compared with control rats. CMS, chronic mild stress.

Cholesterol determination revealed that rats in chronic mild stress exposure produced lower cholesterol levels in the medial prefrontal cortex (P < 0.01, Figure 2a) but not the hippocampus (P > 0.05, Figure 2b) compared to control rats. These findings suggested that the decreased cholesterol levels in the medial prefrontal cortex might be associated with the induction of depressive-like behavior in the chronic mild stress procedure.

### Chronic low dose of cholesterol supplementation prevented depressive-like behavior induced by chronic mild stress

**Figure 3:**
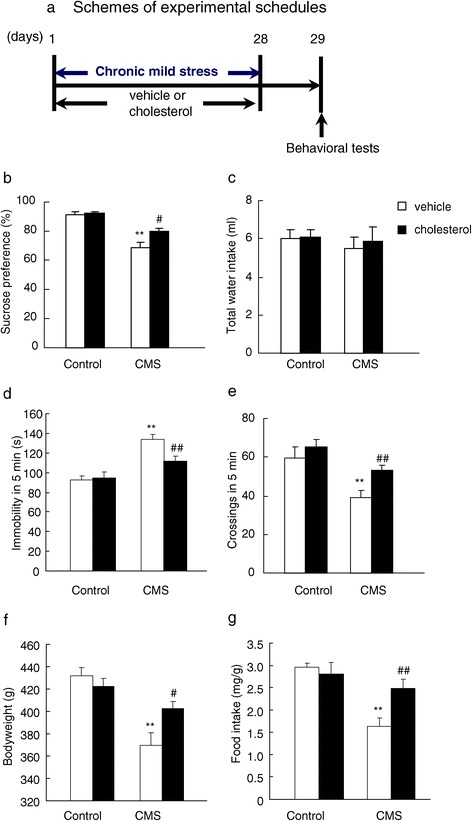
**Chronic cholesterol treatment prevented depressive-like behavior induced by chronic mild stress. (a)** Schemes of experimental schedules, **(b)** sucrose preference, **(c)** total water intake, **(d)** immobility in the forced swim test, **(e)** locomotor activity in the open field test, **(f)** bodyweight, **(g)** food intake. Data are expressed as mean ± SEM (*n* = 8 per group). Differences between control and CMS were assessed using two-way ANOVA followed Tukey’s *post hoc* test. **P < 0.01 compared with control rats. ^#^P < 0.05, ^##^P < 0.01 compared with cholesterol-treated chronic mild stress rats.

We further determine whether pre-treatment with cholesterol before daily stress could prevent the development of depressive-like behavior induced by chronic mild stress. A different set of rats was used in this section (Figure 3a). The results showed that cholesterol added in food for 28 days significantly reversed the depressive-like behavior induced by chronic mild stress as indicated by increased sucrose preference (P < 0.05, Figure 3b, c), decreased immobility (P < 0.01, Figure 3d) in the forced swim test, increased crossings in the open field test (P < 0.01, Figure 3e) and increased bodyweight (P < 0.05, Figure 3f) and food intake (P < 0.01, Figure 3g). The analysis of the sucrose preference data revealed a significant effect of CMS (*F*_1,28_ = 66.20, P < 0.001) and cholesterol (*F*_1,28_ = 6.68, P < 0.05) and a significant CMS × cholesterol interaction (*F*_1,28_ = 5.71, P < 0.05) for sucrose preference. The analysis of immobility in forced swim test showed a significant effect of CMS (*F*_1,28_ = 31.09, P < 0.001) and cholesterol (*F*_1,28_ = 5.69, P < 0.05) and a significant CMS × cholesterol interaction (*F*_1,28_ = 5.30, P < 0.05). Additionally, the data analysis of crossings in the open field test showed a significant effect of CMS (*F*_1,28_ = 15.71, P < 0.001) and cholesterol (*F*_1,28_ = 5.46, P < 0.05) and a significant CMS × cholesterol interaction (*F*_1,28_ = 5.90, P < 0.05). Furthermore, the data analysis of bodyweight changes revealed a significant effect of CMS (*F*_1,28_ = 25.99, P < 0.001) and cholesterol (*F*_1,28_ = 6.09, P < 0.05) and a significant CMS × cholesterol interaction (*F*_1,28_ = 7.05, P < 0.05). Finally, the food intake data analysis showed a significant effect of CMS (*F*_1,28_ = 18.06, P < 0.001) and cholesterol (*F*_1,28_ = 6.41, P < 0.05) and a significant CMS × cholesterol interaction (*F*_1,28_ = 6.57, P < 0.05). These results suggested that cholesterol homeostasis in the central nervous system plays a key role in the depression, especially the stress-related mood disorder. Critical modulation of cholesterol level might be benefit for the treatment and recovery of depressed patients in the clinic.

### Chronic cholesterol treatment increased the cholesterol content in medial prefrontal cortex but not hippocampus

**Figure 4:**
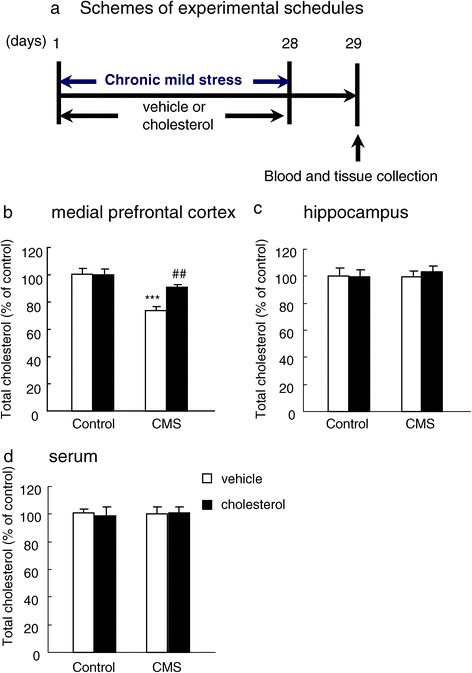
**Chronic cholesterol treatment increased the cholesterol content in medial prefrontal cortex but not hippocampus. (a)** Schemes and timeline of experimental schedules, Total cholesterol content in **(b)** medial prefrontal cortex, **(c)** hippocampus and **(d)** serum. Data are expressed as mean ± SEM (*n* = 8 per group). Differences between control and CMS were assessed using two-way ANOVA followed Tukey’s *post hoc* test. ***P < 0.001 compared with control rats. ^##^P < 0.01 compared with cholesterol-treated chronic mild stress rats.

It is interesting to find that there is a reduction in cholesterol content in medial prefrontal cortex but not in hippocampus upon subjecting rats to chronic mild stress. Therefore, it is important to clarify whether supplementation alters cholesterol content differentially in these two regions. The results showed that chronic cholesterol treatment for 28 days increased cholesterol content in medial prefrontal cortex (Figure 4a,b) of rats in chronic mild stress. The two-way ANOVA analysis showed a significant effect of CMS (*F*_1,28_ = 25.70, P < 0.001) and cholesterol (*F*_1,28_ = 5.40, P < 0.05) and a significant CMS × cholesterol interaction (*F*_1,28_ = 6.43, P < 0.05) for cholesterol content in the medial prefrontal cortex. However, chronic cholesterol treatment for 28 days did not change the cholesterol content in hippocampus (Figure 4c). In addition, the serum cholesterol content was not altered by cholesterol supplementation (Figure 4d). These findings suggested that increase of cholesterol in medial prefrontal cortex, but not hippocampus, by oral cholesterol supplementation is related to the prevention of depressive-like behavior.

### Antagonism of 5-HT1A receptor blocked the behavioral responses of cholesterol in CMS procedure

**Figure 5:**
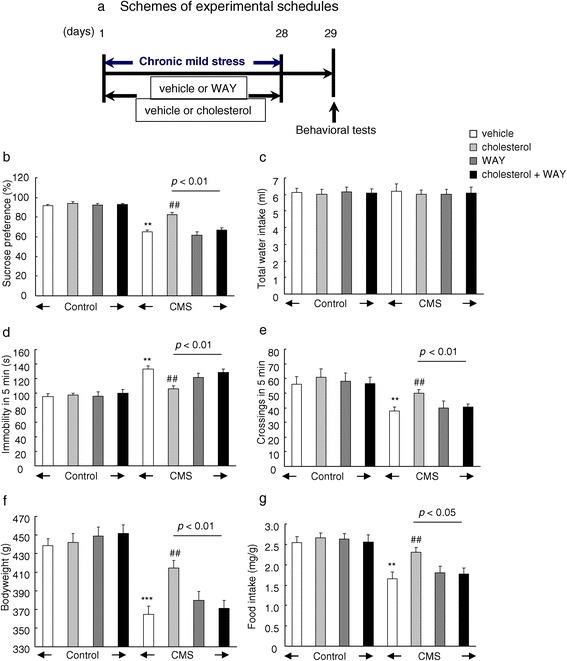
**Antagonism of 5-HT1A receptor blocked the behavioral responses of cholesterol in chronic mild stress procedure. (a)** Schemes of experimental schedules, **(b)** sucrose preference, **(c)** total water intake, **(d)** immobility in the forced swim test, **(e)** locomotor activity in the open field test, **(f)** bodyweight, **(g)** food intake. Data are expressed as mean ± SEM (*n* = 8 per group). Differences between control and CMS were assessed using two-way ANOVA followed Tukey’s *post hoc* test. **P < 0.01, ***P < 0.001 compared with control rats. ^##^P < 0.01 compared with cholesterol-treated chronic mild stress rats.

To further examine the regulatory role of 5-HT system in the relationship between cholesterol dysfunction and depressive symptoms, we exposed eight groups of rats (*n* = 8 per group) either to chronic mild stress or control administration, and cholesterol or 5-HT1A receptor antagonist WAY100635 was microinjected into the medial prefrontal cortex during 28 days (Figure 5a). One day after the last stressor, we measured the depressive-like behavior of these rats. We found that infusion of WAY100635 significantly reversed the behavioral response of supplementation of cholesterol in the chronic mild stress procedure. Chronic cholesterol provision increased the sucrose preference in stress-treated rats (*F*_1,28_ = 20.81, P < 0.001, Figure 5b, c), however, WAY100635 prevented this reversal effects of cholesterol (*F*_1,28_ = 6.51, P < 0.05). Moreover, the reduction of immobility in the forced swim test (*F*_1,28_ = 11.69, P < 0.01, Figure 5d) and crossings in the open field test (*F*_1,28_ = 5.19, P < 0.05, Figure 5e) was also blocked by WAY100635. Similarly, the increased bodyweight (*F*_1,28_ = 10.73, P < 0.01, Figure 5f) and food intake (*F*_1,28_ = 5.38, P < 0.05, Figure 5g) was both reversed by WAY100635 microinjection. This finding suggested that cholesterol may have an impact on the sensitivity of the 5-HT receptors in the interactions between stress and depression.

## Discussion

In the current investigation, we found that chronic mild stress induced depressive-like behavior in rats associated with decreased cholesterol levels in the medial prefrontal cortex. However, pre-treatment with cholesterol before daily stress could prevent the development of depressive-like behavior induced by chronic mild stress. Finally, intra-mPFC infusion of 5-HT1A receptor antagonist WAY100635 significantly reversed the behavioral response of supplementation of cholesterol in the chronic mild stress procedure. This data suggested that regulation of 5-HT1A receptor participated in the development of depression through control of central cholesterol in rats. In patients with coronary artery disease, the prevalence of several diseases has been increased, such as type 2 diabetes mellitus [35,36] and symptoms of depression [37], leading to increased risk for further cardiac events after acute coronary syndrome. Moreover, about 17%–27% of patients with coronary artery disease have major depression, and a significantly larger percentage have subsyndromal symptoms of depression [38]. Depression has been linked to higher health care costs and to worse outcomes in patients with coronary artery disease [39,40]. Selective serotonin reuptake inhibitors (SSRIs) are the first-line antidepressants among patients with coronary artery disease with a lower risk of death/recurrent myocardial infarction [41].

Since low and higher level of serum total cholesterol is a stable feature in some persons with recurrent major depression, the level of total cholesterol in blood samples in patients with major depression has been widely used to estimate the risk of suicidal behavior in the presence of depressive disorder [42]. Consistent with previous findings that serum cholesterol was decreased in depressed patients, our results showed that the cholesterol levels in the medial prefrontal cortex were significantly decreased in rats that were exposed to chronic mild stress for a long period. According to this data, we therefore proposed that the reduced central levels of cholesterol might be related with the onset of depressive-like behavior in rats. However, not all studies suggested a relationship between lower cholesterol and depression. There are some evidences showed that the elevated cholesterol levels were seen in depressed patients [43]. Furthermore, there are strong evidences indicated that serum cholesterol level was increased in patients with coronary artery diseases. However, we focused on the changes of cholesterol level in the central nervous system in our current study. Our data showed cholesterol was decreased in the medial prefrontal cortex, but not in the hippocampus, of the brain in rats exposed to chronic mild stress, which suggested that the alteration of cholesterol in specific brain region might play a different role in the regulation of mood by specific downstream signal pathway. There are many differences between medial prefrontal cortex and hippocampus beyond the number of 5-HT1A receptor. It has been evidenced that chronic mild stress significantly decreased PSD95, a key synaptic protein that co-localizes with NMDA receptors at synapses, inhibited mTOR function by decreasing phosphorylated p70s6k and rps6 in the medial prefrontal cortex but not hippocampus [44]. Induction of PSD-95 is consistent with increased synapse formation and function. mTOR signaling pathways have been implicated in the increase in synaptic plasticity and new spine formation. Therefore, we raised the possibility that these post-synaptic signal pathways in the medial prefrontal cortex might play a central role in depression symptoms after cholesterol treatment.

The relationship between cholesterol levels, depressive behavior and serotonergic function could be explained that the changed levels of cholesterol are directly responsible for alterations in serotonergic function in depression. Cholesterol is an important membrane lipid and is essential in regulating the properties of cell membranes in mammalian cells organization, dynamics, function, and sorting [45,46].

Several studies showed that chronic administration of stress hormone, such as glucocorticoid corticosterone and adrenocorticotropic hormone, increases muscle tissues and plasma cholesterol in animals [47,48]. However, there is no direct evidence revealed the effects of stress on the levels of cholesterol in the central nervous system. Additionally, other abnormalities related to lipid homeostasis described in depressed patients include an increase in the activity of enzymes involved in lipid oxidation and peroxidation [49], lower vitamin E concentrations [50], and lower serum high-density lipoprotein-cholesterol (HDL-C) levels [51]. Previous study showed that simvastatin, a cholesterol-lowering agent, increased the expression of brain-derived neurotrophin factor (BDNF) and vascular endothelial growth factor (VEGF) in the dentate gyrus (DG) of the hippocampus in rats after traumatic brain injury, suggesting an improvement of cognitive function [52]. Furthermore, pravastatin administration promotes neurological recovery in ischemic stroke animals and induces neurogenesis in the DG and subventricular zone (SVZ), and increases the number of migration cells in the striatum [53]. Cholesterol exists in plasma membrane has been evidence to play an important role in neuronal differentiation, synaptogenesis and axonal guidance [54]. These findings raised the possibility that regulation of cholesterol might play a role on neuroprotection and neurogenesis, which is associated with the stress and depressive disorder. Moreover, serotonin plays a direct and acute regulatory role in activity-dependent hippocampal neurogenesis [55]. Therefore, it could be possible that understanding of cholesterol on the regulation of neurogenesis might offer preventive therapeutic opportunities in depression. Additionally, the changes of cholesterol in serum affect neurotransmission in the central nervous system [10]. The specific neurotransmitter by which cholesterol regulated in the pathophysiology of depression needs further clarification.

Chronic mild stress significantly reduced 5-HT1A receptor in the prefrontal cortex in rats [56]. Additionally, early-life stress increased 5-HT1A receptor mRNA expression in the amygdala, and reduced its expression in the dorsal raphé nucleus [57]. These findings suggest that stress induces persistent changes in 5-HT1A receptor and expression in major brain regions involved in the development of stress-related psychiatric disorders. In our current study, we found that intra-mPFC infusion of 5-HT1A receptor antagonist WAY100635 significantly reversed the behavioral response of supplementation of cholesterol in the chronic mild stress procedure. Previous evidence also showed that membrane cholesterol plays a key role in the stability of the human serotonin (1A) receptor [58]. It can be presumed that cholesterol binds 5-HT1A receptors [59] and therefore induces translocation to increase serotonin release to counteract depression. Therefore, further studies are required to clarify the cellular mechanisms through which this relationship between cholesterol and depression works.

## Conclusion

In summary, chronic supplementation of cholesterol by food reversed the depressive-like behavior induced by chronic mild stress in rats. Furthermore, pre-injection with 5-HT1A receptor antagonist WAY100635 into the medial prefrontal cortex blocked the treatment effects of cholesterol on the reversal of behavioral response. The present finding revealed that cholesterol may have an impact on the sensitivity of the 5-HT receptors in the interactions between stress and depression. The treatment benefits of cholesterol could be acting through modulation of the brain serotonin system especially in the medial prefrontal cortex.
